# Safety and immunogenicity of an upper-range release titer measles-mumps-rubella vaccine in children vaccinated at 12 to 15 months of age: a phase III, randomized study

**DOI:** 10.1080/21645515.2018.1502527

**Published:** 2018-08-29

**Authors:** The MMR-162 Study Group

**Keywords:** MMR vaccine, measles, mumps, rubella, safety, release titer

## Abstract

The titer of live attenuated viral vaccines, such as MMR vaccines, varies between batches and over the shelf-life of a batch, with the highest titer expected at batch release. As higher titers may theoretically lead to increased reactogenicity, we compared the safety profile of an upper-range release titer MMR-RIT lot with commercial MMR II lots in a phase III, randomized, controlled study (NCT02184572). We vaccinated 1736 children with MMR-RIT (N = 1164) or MMR II (N = 572), both administered as first doses with varicella, hepatitis A, and pneumococcal conjugate vaccines at 12–15 months of age. The incidence of fever 5–12 days post-vaccination was comparable following MMR-RIT and MMR II vaccination: 4.2% vs 3.1% (difference: 1.1%) for fever > 39.0°C and 18.2% vs 17.1% (difference: 1.1%) for fever ≥ 38.0°C, which met the primary objective. Two cases of febrile convulsions (one considered vaccination-related) were reported within 43 days post-MMR-RIT. During Days 0–42, rashes were reported for 24.4% (MMR-RIT) and 27.4% (MMR II) of children; measles/rubella-like rashes for 5.8% and 4.7%, respectively. Measles-like illnesses were reported for 1.5% (MMR-RIT) and 0.9% (MMR II) of children 5–12 days post-vaccination. One serious adverse event, immune thrombocytopenic purpura following MMR II vaccination, was considered vaccination-related. Immune responses were similar in both groups. In summary, the safety profile of an upper-range release titer MMR-RIT lot was in line with that of commercial MMR II lots, with similar rates of fever and other MMR-specific symptoms and low rates of measles-like illnesses reported with both vaccines.

## Introduction

The introduction of vaccines against measles, mumps, and rubella in childhood immunization programs has led to considerable reductions in the incidence of the diseases and the associated morbidity and mortality caused by these viruses.^1–3^ However, occasional measles, mumps, and rubella outbreaks still occur, often caused or aggravated by sub-optimal vaccination coverage in certain areas or population groups.^3–7^ Sustained high vaccination coverage is therefore crucial for optimal disease prevention.^8–11^ Combined live attenuated measles, mumps, and rubella (MMR) vaccines help simplify childhood immunization schedules and therefore facilitate achieving high coverage.^12^

The only combined MMR vaccine currently licensed in the United States (US), MMR II (*M-M-R II*, Merck & Co Inc.), is recommended as a two-dose schedule, with a first dose given at 12–15 months and a second at 4–6 years of age.^13^ A third dose has recently been recommended in persons at increased risk of acquiring mumps during an outbreak.^14^ Considering the importance of maintaining high vaccination coverage with at least two vaccine doses, an interruption in the MMR II vaccine supply could have serious public health consequences. The availability of a second MMR vaccine in the US could help mitigate this public health risk.^12^ GSK’s MMR vaccine, MMR-RIT (*Priorix*), is licensed in more than 100 countries outside of the US for use in children 9 months or older and is typically given as a two-dose schedule.^15,16^

The concentration of viruses in live attenuated viral vaccines (or so-called titer) varies slightly between vaccine batches at release, as a result of the manufacturing process. Titers will typically decline over the shelf-life of a batch. While batches with a low titer need to be tested to safeguard efficacy of the vaccine at the end of shelf-life, it is equally important to assess the safety of batches with a titer as high as what may be expected when the batch is released.^17–20^ As higher viral titers in a vaccine lot may theoretically lead to increased reactogenicity and safety concerns, the current study aimed to evaluate whether the safety profile of the MMR-RIT vaccine at an upper-range release titer (not tested thus far) was clinically acceptable. We therefore compared this upper-range release titer MMR-RIT lot with commercial lots of MMR II vaccine both administered in children 12–15 months of age together with varicella, hepatitis A, and pneumococcal conjugate vaccines, reflecting the standard of care in the US.

MMR vaccines have been found to be well tolerated and rarely linked to serious adverse events (SAEs).^15,21,22^ However, they are associated with a risk of fever and febrile convulsions 7–10 days after first vaccination (mostly due to the replication of the measles component).^22–30^ MMR vaccination may also cause transient rashes (due to the measles and rubella components), usually appearing 1–2 weeks after vaccination; swelling of the parotid or other salivary glands (due to the mumps component); and other symptoms frequently observed for pediatric vaccines, including injection site reactions.^15,22,24,30^ We therefore evaluated the incidence of fever 5–12 days after vaccination as primary objective, and the occurrence of measles-like illness during the same time period, febrile convulsions, rashes, parotid/salivary gland swelling, and other safety outcomes, as well as immunogenicity as secondary objectives.

## Results

### Study participants

**Figure 1.**
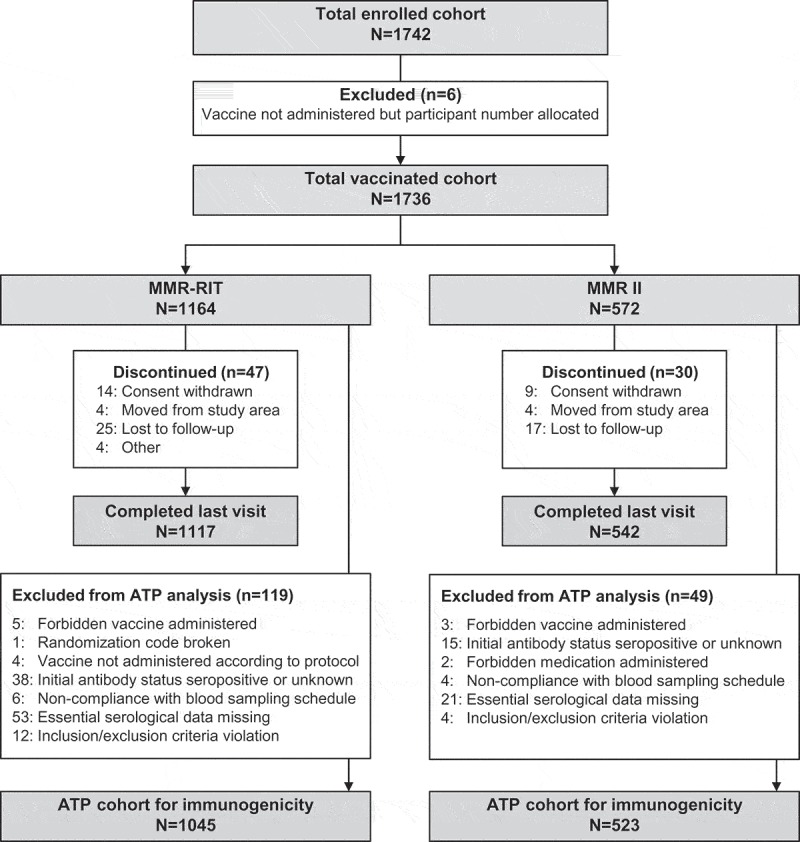
Participant flow diagram. N, number of children; n, number of children in a given category; ATP, according-to-protocol.

We enrolled 1742 children of whom 1736 were included in the total vaccinated cohort; 1164 received the upper-range release titer MMR-RIT vaccine (MMR-RIT group) and 572 received one of two commercial lots of MMR II control vaccine (MMR II group). We used two different MMR II lots to minimize variability in titers of available lots and thereby obtain more representative data. Data for the two MMR II lots were pooled for all analyses. A total of 1659 children completed the study and 1568 were included in the according-to-protocol (ATP) cohort for immunogenicity. Reasons for withdrawal from the study and exclusion from the ATP cohort for immunogenicity are provided in Figure 1. Demographic characteristics were similar between the two groups. The mean age at baseline was 12.3 months and approximately 63% of children were enrolled in the US, 14% in Estonia, 13% in Finland, and 11% in Taiwan (Table 1).

**Table 1.** Demographic characteristics of study participants at baseline (total vaccinated cohort).

Characteristic	MMR-RIT N = 1164	MMR II N = 572
Age in months, mean (SD)	12.3 (0.7)	12.3 (0.7)
Female gender, n (%)	551 (47.3)	270 (47.2)
Race, n (%)		
Caucasian/European heritage	808 (69.4)	385 (67.3)
Asian heritage	170 (14.6)	81 (14.2)
African heritage/African American	64 (5.5)	38 (6.6)
Other	122 (10.5)	68 (11.9)
Country, n (%)		
Estonia	160 (13.7)	80 (14.0)
Finland	147 (12.6)	73 (12.8)
Taiwan	123 (10.6)	62 (10.8)
US	734 (63.1)	357 (62.4)

N, number of children in the total vaccination cohort in each group; n (%), number (percentage) of children in a given category; SD, standard deviation.

### Safety and reactogenicity

**Figure 2.**
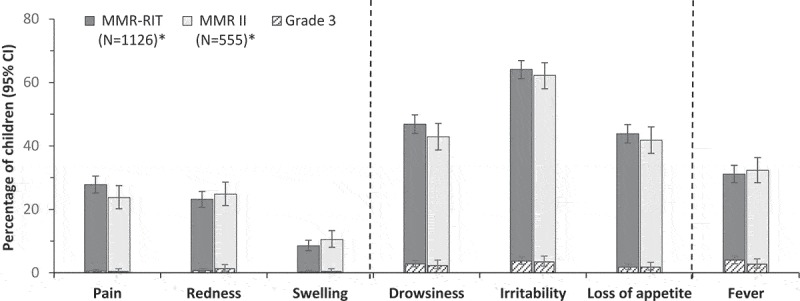
Incidence of solicited injection site symptoms (Days 0–3), general symptoms (Days 0–14), and fever (Days 0–42) (total vaccinated cohort). CI, confidence interval; N, number of children with documented dose (*for pain, redness, and swelling: N = 1123 for MMR-RIT and N = 553 for MMR II). Fever was defined as a temperature ≥ 38.0°C/100.4°F. Grade 3 intensity was defined as crying when the limb was moved or the limb was spontaneously painful (pain), diameter > 20 mm (redness, swelling), preventing normal activity (drowsiness), crying inconsolably or preventing normal activity (irritability), not eating at all (loss of appetite), temperature > 39.5°C/103.1°F (fever). Injection site symptoms were those recorded at the MMR injection site.

**Figure 3.**
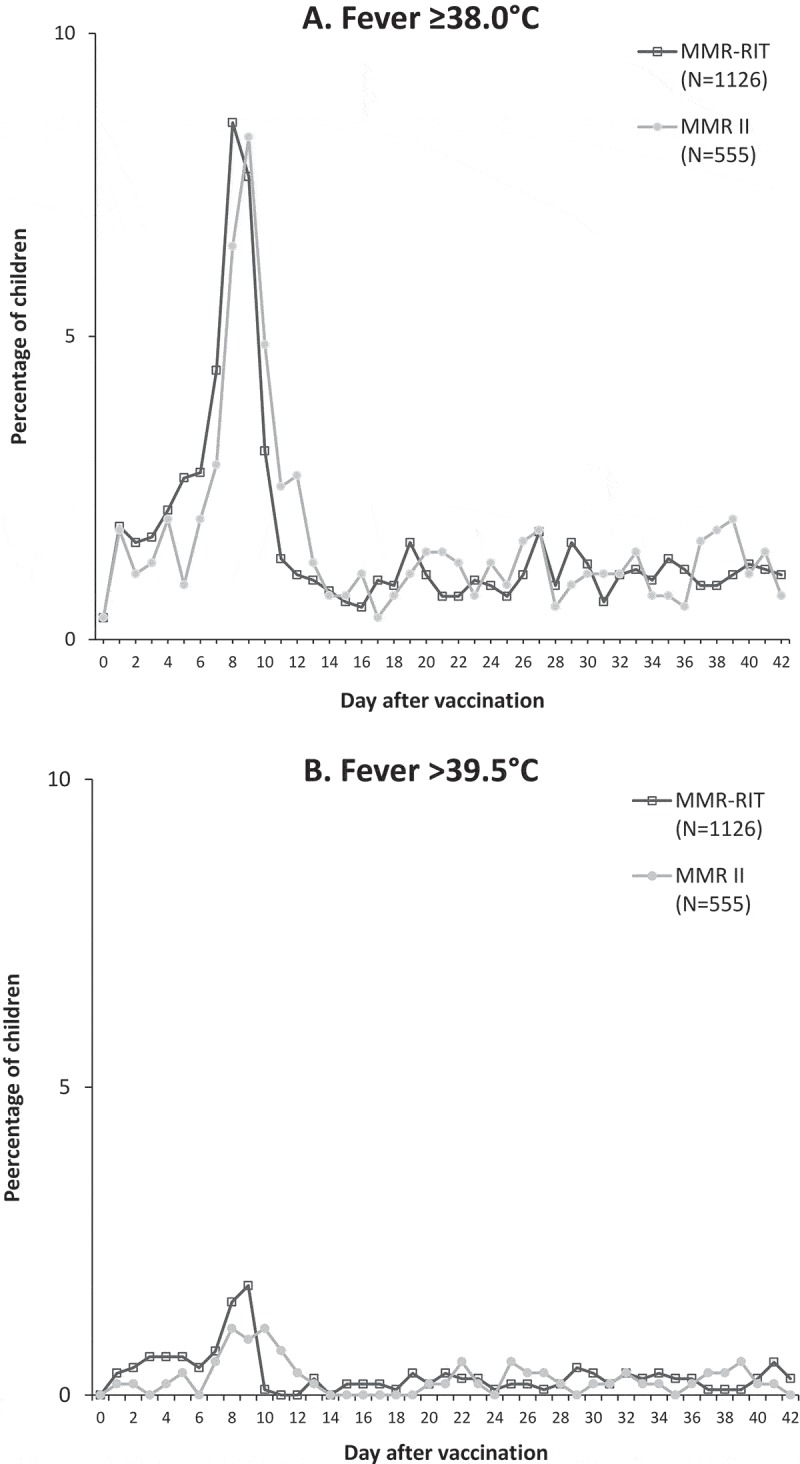
Prevalence of fever ≥ 38.0°C (A) and > 39.5°C (B) during Days 0–42 post-vaccination (total vaccinated cohort). N, number of children with documented dose.

During the 5–12-day post-vaccination period, the incidences of fever > 39.0°C and ≥ 38.0°C were 4.2% and 18.2% in children vaccinated with the upper-range release titer MMR-RIT lot, and 3.1% and 17.1% in children who received the commercial MMR II lots (Table 2). The co-primary objectives to demonstrate the safety profile in terms of fever > 39.0°C and ≥ 38.0°C were met: the upper limits of the two-sided standardized asymptotic 95% confidence intervals (CIs) for the group differences (MMR-RIT minus MMR II) in the incidence of fever were 2.89% (for fever > 39.0°C) and 4.85% (for fever ≥ 38.0°C), and thus below the predefined limits of 5% and 10%, respectively (Table 2). The incidence of fever reported as solicited general symptom during the 43-day post-vaccination period was also similar between groups, with 31.1% of MMR-RIT and 32.3% of MMR II-vaccinated children reporting fever ≥ 38.0°C, and 4.0% and 2.7% reporting grade 3 fever (> 39.5°C) (Figure 2 and Supplemental table S1). Medical advice for fever was sought for 13.1% and 10.1% of children in the MMR-RIT and MMR II groups, respectively. The peak prevalence in fever occurred between Days 7 and 10 after vaccination in both groups (Figure 3).

**Table 2.** Difference between groups in the incidence of fever during Days 5–12 post-vaccination (total vaccinated cohort).

	MMR-RIT N = 1126		MMR II N = 555		
Fever	n	%	n	%	Difference in % MMR-RIT minus MMR II (95% CI)
Temperature > 39.0°C	47	4.2	17	3.1	1.11 (−0.93, 2.89*)
Temperature ≥ 38.0°C	205	18.2	95	17.1	1.09 (−2.89, 4.85*)

N, number of children with documented dose; n and %, number and percentage of children reporting fever (temperature > 39.0°C or ≥ 38.0°C) at least once during the 5–12-day post-vaccination period; CI, confidence interval.

*Criteria to demonstrate co-primary objectives: upper limit of the 2-sided standardized asymptotic 95% CI for the group difference (MMR-RIT minus MMR II) in incidence of fever within 5–12 days after vaccination was ≤ 5% (for temperature > 39.0°C) or ≤ 10% (for temperature ≥ 38.0°C).

Solicited injection site symptoms (recorded during Days 0–3) and the general symptoms drowsiness, irritability, and loss of appetite (recorded during Days 0–14) were reported at similar rates after MMR-RIT and MMR II vaccination, with no more than 3.7% of children reporting severe (grade 3) symptoms (Figure 2 and Supplemental table S1).

MMR vaccination is associated with febrile convulsions, and may cause rashes and swelling of the parotid and other salivary glands.^15,22,24,30^ The incidence of these MMR-specific solicited general symptoms recorded during Days 0–42 was similar between groups. Febrile convulsions were reported for two (0.2%) MMR-RIT-vaccinated children. One case occurred 7 days after MMR-RIT vaccination; it was classified as simple febrile convulsion/seizure with Brighton Collaboration level 1 and grade 3 intensity, and was considered vaccination-related. The other case occurred 36 days after MMR-RIT vaccination, was classified as simple febrile convulsion/seizure, and was not considered vaccination-related by the investigator. There were no reports of parotid/salivary gland swelling. Rashes were reported for 24.4% of MMR-RIT and 27.4% of MMR II-vaccinated children. Approximately two thirds of the rashes were localized and occurred without fever. Measles/rubella-like rashes were reported for 5.8% and 4.7% of children; varicella-like rashes (as a possible consequence of the co-administered varicella vaccine) for 3.6% and 4.0% (Table 3). Medical advice for rashes was sought for 11.2% and 12.4% of children in the MMR-RIT and MMR II groups, respectively.

**Table 3.** Incidence of post-vaccination rashes (Days 0–42) and measles-like illnesses (Days 5–12) (total vaccinated cohort).

	MMR-RIT N = 1126*		MMR II N = 555*	
Symptom	n	% (95% CI)	n	% (95% CI)
**Rash**				
Any	275	24.4 (21.9, 27.0)	152	27.4 (23.7, 31.3)
Localized	185	16.4 (14.3, 18.7)	98	17.7 (14.6, 21.1)
Generalized	109	9.7 (8.0, 11.6)	64	11.5 (9.0, 14.5)
With fever	100	8.9 (7.3, 10.7)	48	8.6 (6.5, 11.3)
Grade 3	22	2.0 (1.2, 2.9)	8	1.4 (0.6, 2.8)
Measles/rubella-like	65	5.8 (4.5, 7.3)	26	4.7 (3.1, 6.8)
Varicella-like	40	3.6 (2.6, 4.8)	22	4.0 (2.5, 5.9)
**Measles-like illness**				
Any	18	1.5 (0.9, 2.4)	5	0.9 (0.3, 2.0)

N, number of children with documented dose (*for measles-like illnesses: N = 1164 for MMR-RIT and N = 572 for MMR II); n and %, number and percentage of children reporting the specified type of rash or measles-like illness at least once; CI, confidence interval. Grade 3 intensity was defined as > 150 lesions (measles/rubella or varicella-like rash) or rash preventing normal activity (other rashes). Measles-like illness was defined as the occurrence of the following signs or symptoms in the absence of another confirmed diagnosis: temperature ≥ 38.0°C and maculopapular rash (including measles/rubella-like rash) during Days 5–12 post-vaccination, and at least one of the following signs or symptoms: cough, runny nose, conjunctivitis, or diarrhea.

Children with fever (≥ 38.0°C) and a potential measles-like rash occurring 5–12 days post-vaccination were further examined for the presence of other symptoms associated with measles to assess the occurrence of measles-like illness. Measles-like illnesses were reported for 1.5% of MMR-RIT and 0.9% of MMR II-vaccinated children between Days 5 and 12 post-vaccination (Table 3).

A total of 51.4% (95% CI: 48.5%, 54.3%) and 48.4% (44.3%, 52.6%) of children in the MMR-RIT and MMR II groups, respectively, reported unsolicited adverse events (AEs) during the 43-day post-vaccination period; upper respiratory tract infection (9.5% and 12.8%) and diarrhea (8.2% and 8.0%) occurred most frequently. Grade 3 unsolicited AEs were reported for 4.6% (3.5%, 6.0%) of MMR-RIT and 3.8% (2.4%, 5.8%) of MMR II-vaccinated children. Unsolicited AEs considered as vaccination-related were reported for 4.6% (3.5%, 6.0%) and 4.0% (2.6%, 6.0%) of children.

Throughout the study, 39 SAEs were reported in 24 (2.1%) MMR-RIT-vaccinated children and 12 SAEs in 9 (1.6%) MMR II-vaccinated children. One SAE, immune thrombocytopenic purpura diagnosed two days after MMR II vaccination, was considered vaccination-related by the investigator. The disorder was still ongoing at the last contact with the child (approximately eight months after onset) but the child was lost to follow-up thereafter. There were no deaths in this study.

New onset chronic diseases (NOCDs) were reported in 2.5% (95% CI: 1.7%, 3.6%) of MMR-RIT and 1.9% (1.0%, 3.4%) of MMR II-vaccinated children. None of these were considered vaccination-related. The most frequently observed NOCDs were atopic dermatitis in the MMR-RIT group (9 children, 0.8%), and allergic dermatitis in the MMR II group (3 children, 0.5%). Throughout the study, 14.3% (12.3%, 16.4%) of MMR-RIT and 9.6% (7.3%, 12.3%) of MMR II-vaccinated children had an AE that prompted an emergency room (ER) visit, with (acute) otitis media (MMR-RIT: 23 children, 1.9%; MMR II: 9 children, 1.5%), upper respiratory tract infection (MMR-RIT: 21 children, 1.8%; MMR II: 5 children, 0.9%), and fever (MMR-RIT: 14 children, 1.2%; MMR II: 7 children, 1.2%) being among the most common causes for ER visits.

### Immunogenicity

Day 42 seroresponse rates were 99.0% and 96.5% for measles, 99.4% and 97.9% for mumps, and 95.7% and 98.3% for rubella after MMR-RIT and MMR II vaccination, respectively (Table 4). Day 42 antibody geometric mean concentrations (GMCs) exceeded the assay cut-offs for seropositivity by at least 10-fold for the three antigens. Antibody GMCs for measles and mumps were in similar ranges between groups while anti-rubella antibody GMCs tended to be lower in MMR-RIT compared to MMR II-vaccinated children (Table 4).

**Table 4.** Seroresponse rates and geometric mean concentrations for antibodies to measles, mumps, and rubella 42 days after vaccination in initially seronegative children (ATP cohort for immunogenicity).

	MMR-RIT			MMR II		
Antibody (unit)	N	Seroresponse rate, % (95% CI)	Antibody GMC (95% CI)	N	Seroresponse rate, % (95% CI)	Antibody GMC (95% CI)
Measles (mIU/mL)	1043	99.0 (98.2, 99.5)	2751.9 (2618.3, 2892.2)	521	96.5 (94.6, 97.9)	3133.3 (2878.6, 3410.6)
Mumps (EU/mL)	964	99.4 (98.7, 99.8)	86.0 (82.0, 90.3)	483	97.9 (96.2, 99.0)	82.6 (76.5, 89.2)
Rubella (IU/mL)	1043	95.7 (94.3, 96.8)	45.0 (42.8, 47.2)	521	98.3 (96.7, 99.2)	66.8 (62.3, 71.7)

ATP, according-to-protocol; N, number of children with available results (lower for mumps assessment because the laboratory performing the mumps enzyme-linked immunosorbent assay, Pharmaceutical Product Development, Inc., was no longer available to test samples of children enrolled near the end of the enrollment period); CI, confidence interval; mIU, milli international units; EU, enzyme-linked immunosorbent assay units; IU, international units; seroresponse rate, % of children who were seronegative before vaccination and had a post-vaccination antibody concentration ≥ 200 mIU/mL for measles, ≥ 10 EU/mL for mumps, and ≥ 10 IU/mL for rubella. Cut-offs for seropositivity before vaccination: 150 mIU/mL for measles, 5 EU/mL for mumps, and 4 IU/mL for rubella.

## Discussion

In this randomized study on more than 1700 children, we compared an upper-range release titer MMR-RIT vaccine lot with commercial lots of MMR II vaccine and showed that both vaccines had a comparable reactogenicity and safety profile when administered as a first dose to children 12–15 months of age, together with varicella, hepatitis A, and pneumococcal conjugate vaccines, according to the standard of care. Both vaccines also induced a similar immune response.

It is important to evaluate the safety and reactogenicity of live attenuated viral vaccines at an upper-range release titer (i.e., a titer at the upper end of what may be expected at batch release) because higher viral titers in a vaccine may theoretically result in a higher rate of AEs.

We assessed fever (≥ 38.0°C and > 39.0°C) during the 5–12-day post-vaccination period as the primary objective in our study (because of the known association between a first dose of measles-containing vaccines and fever 7–10 days post-vaccination^15,22,27,29^), and demonstrated a comparable incidence of fever with the upper-range release titer MMR-RIT lot and the commercial MMR II lots used in this study. For both vaccines, the peak in fever prevalence occurred during this 5–12-day time window, more specifically between Days 7 and 10, which coincides with the peak in measles viral replication.^31^ The incidence of fever reported over the 43-day post-vaccination period was also similar for both vaccines, and was in line with the incidence observed in previous studies with both vaccines conducted in various countries.^15,32–38^

Due to the increased fever rates, a first dose of measles-containing vaccines is also associated with a risk of febrile convulsions 7–10 days post-vaccination, with an estimated MMR-attributable risk of 1:3000–1:4000.^22,23,25,26^ In our study, two (0.2%) cases of febrile convulsions were reported within 43 days after MMR-RIT vaccination, one of which occurred during the known risk period and was considered vaccination-related.

We also evaluated the occurrence of other measles symptoms associated with the measles viral replication by defining measles-like illness as a safety endpoint. To address the hypothetical risk that MMR-RIT vaccine at an upper-range release titer may induce a measles-like illness, children presenting with fever or a rash during the 5–12-day post-vaccination period were examined further for the co-occurrence of both fever and a maculopapular (measles/rubella-like) rash, and at least one other symptom typically associated with measles illness such as cough, runny nose, conjunctivitis, or diarrhea. Measles-like illnesses during the 5–12-day post-vaccination period were reported infrequently (≤ 1.5%) in both groups.

A transient rash typically occurs in approximately 5% of children after MMR vaccination.^15,22,24,30^ In our study, rashes were reported with similar frequencies in both groups and most had no impact on daily activity. Mumps-containing vaccines may cause swelling of the parotid gland in 1%–2% of vaccine recipients (parotitis being the most common manifestation of mumps illness).^15,24,30^ In our study, there were no reports of swelling of the parotid or other salivary glands.

The incidence of solicited symptoms at the MMR injection site within 4 days after either MMR-RIT or MMR II vaccination was similar, with pain being reported more frequently (23.7%–27.8%) than in previous studies in various countries (≤ 15.8%),^15,32–34,37,38^ but at similar rates as in a more recent phase II study in the US (24.5%–28.0%).^36^ In that phase II study, MMR vaccines were co-administered with varicella, hepatitis A, and pneumococcal conjugate vaccines, like in the US children enrolled in our study. Injection site reactions after vaccination with pneumococcal conjugate vaccines are relatively common.^39^ Even though in our study the co-administered vaccines were given at different injections sites and through different routes, a higher rate of pain at the pneumococcal injection site may have influenced the assessment of pain at the other injection sites by the parents/legally acceptable representatives (LARs). In-depth education of the parents/LARs on the use of diary cards may also have prompted an increased reporting rate. Most importantly, injection site pain was mainly mild and was reported at similar rates in both groups.

There was no imbalance between the groups in the incidences of unsolicited AEs, NOCDs, AEs requiring an ER visit, or SAEs. One SAE, immune thrombocytopenic purpura in the MMR II group, was considered vaccination-related. The outcome of this SAE is unknown because the child was lost to follow-up and the disorder was still ongoing at the last contact. MMR vaccination in children has been shown to be associated with thrombocytopenic purpura (with an incidence ranging from 0.087 to 4 cases per 100,000 MMR vaccine doses), but most cases are self-limited and non-life threatening.^40,41^

In our study, both vaccines induced a similar immune response, with 95.7%–99.0% of children showing a seroresponse against the different vaccine antigens, in line with what was shown in previous studies.^15,36^ GMCs for antibodies against rubella virus were numerically lower in the MMR-RIT than in the MMR II group but the clinical relevance of this is unknown considering the 95.7% seroresponse rate among these children.

Our study has some limitations. Although the sample size of our study was relatively large, it may have precluded the detection of rare AEs. In addition, except for the primary objective, all other endpoints were descriptive with no adjustment for multiplicity. The choice of commercial lots of MMR II rather than MMR-RIT as comparator in the current study could be considered as a study limitation. However, the choice of MMR II as comparator in the current study was guided by regulatory constraints to assess the safety versus an established registered comparator in the countries where the study was conducted. Another limitation may be that the exact viral titer of the MMR II comparator lots was not known. However, MMR II titers likely declined over the course of the study and would on average have been lower than the titer of the upper-range release titer MMR-RIT lot.

In summary, the fever profile of MMR-RIT vaccine at an upper-range release titer was comparable to that of commercial lots of MMR II, when administered as a first dose to 12–15-month-old children, together with varicella, hepatitis A, and pneumococcal conjugate vaccines, which are part of the routine pediatric immunization schedule in the US. No safety concerns were identified in this study, and the reactogenicity of the upper-range release titer MMR-RIT vaccine was in line with that of the MMR II vaccine, with similar rates of MMR-specific symptoms and a low rate of measles-like illnesses reported with both vaccines. Immune responses induced by single doses of both vaccines were comparable. Together with the large amount of data from other clinical trials and post-marketing studies, this supports the use of MMR-RIT as a valid second MMR vaccine in the US, should it be licensed for use there.

**Figure 4.**
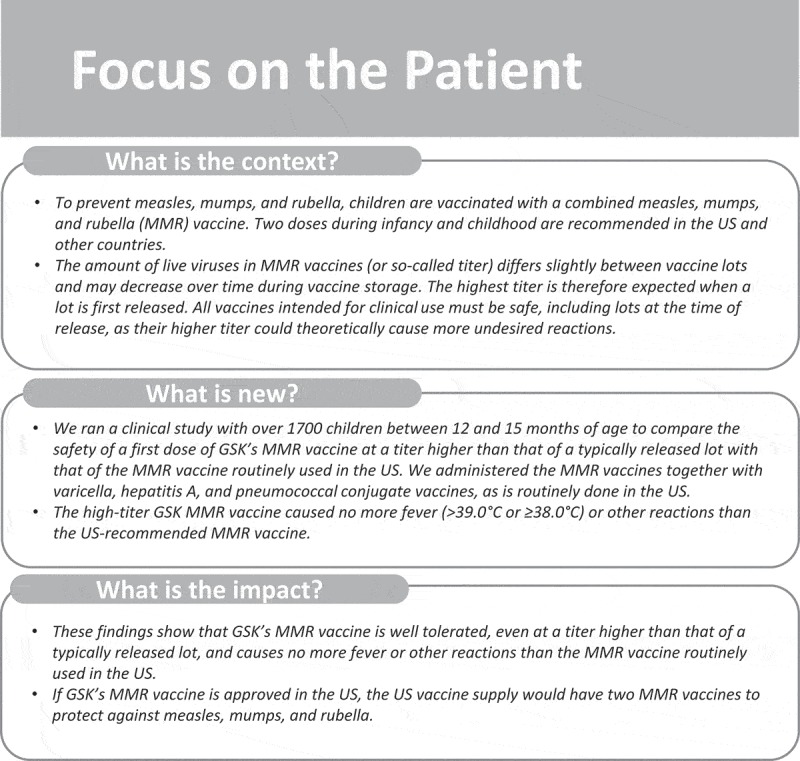
Focus on the patient.

A summary of the clinical relevance of the research, aimed to be shared with patients by health care providers, is represented in Figure 4.

## Patients and methods

### Study design, participants, and vaccines

This phase III, observer-blind, randomized, controlled study (ClinicalTrials.gov: NCT02184572) with three parallel groups was performed at 100 centers in Estonia, Finland, Taiwan, and the US (including Puerto Rico) between 25 August 2014 and 22 December 2015. The study was conducted according to the principles of Good Clinical Practice, the Declaration of Helsinki, and all applicable regulatory requirements. Independent ethics committees or institutional review boards reviewed and approved the protocol and other study-related documents. A summary of the protocol is available at https://www.gsk-clinicalstudyregister.com/study/115650. An independent data monitoring committee monitored the safety of the enrolled children.

**Figure 5.**
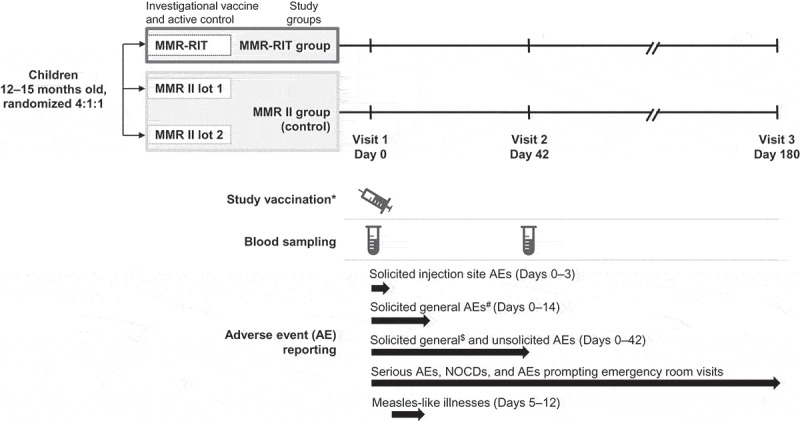
Study design. *Study vaccines were co-administered with varicella and hepatitis A vaccines in all children, and with the 13-valent pneumococcal conjugate vaccine in children in the United States. ^#^Drowsiness, irritability, and loss of appetite. ^$^Fever, rash, parotid/salivary gland swelling, and febrile convulsions. NOCDs, new onset chronic diseases.

The study consisted of three visits (on Days 0, 42, and 180) (Figure 5). Healthy children aged 12–15 months were randomized using a blocking scheme (4:1:1) to receive a single dose of either an upper-range release titer lot of MMR-RIT vaccine or one of two commercial lots of MMR II control vaccine (two different lots were used per vaccine shipment; multiple lots were used over the study). MMR vaccines were administered subcutaneously in the left triceps. The viral titer of the MMR-RIT lot used in this study exceeded that of a typically released lot; it contained 10⁴.⁵ cell culture infectious dose 50 (CCID₅₀) live attenuated measles virus (Schwarz strain), 10⁵.⁷ CCID₅₀ live attenuated mumps virus (RIT4385 strain), and 10⁴.⁴ CCID₅₀ live attenuated rubella virus (Wistar RA 27/3 strain). Titers were measured before study start (on 18 October 2013) and vaccine doses were kept frozen at −15°C to −25°C until use to maintain the titer as close as possible to that measured on that date. The actual titer of the MMR II lots was not measured and was assumed to be in line with the manufacturer’s product information: ≥ 10^3.0^ tissue culture infectious dose 50 (TCID_50_) live attenuated measles virus (Enders’ Edmonston strain), ≥ 10^4.1^ TCID_50_ live attenuated mumps virus (Jeryl Lynn strain), and ≥ 10^3.0^ TCID_50_ live attenuated rubella virus (Wistar RA 27/3 strain).

All children received single doses of varicella vaccine (*Varivax*, Merck & Co Inc.), subcutaneously in the right triceps, and hepatitis A vaccine (*Havrix*, GSK), intramuscularly in the right anterolateral thigh, concomitantly with MMR vaccine. Children enrolled in the US also received the fourth dose of 13-valent pneumococcal conjugate vaccine (PCV13; *Prevnar 13/Prevenar 13*, Pfizer Inc.), intramuscularly in the left anterolateral thigh. At the end of the study (and outside of study procedures), a second dose of varicella and hepatitis A vaccines was offered to children enrolled in selected non-US countries if local health departments did not routinely provide varicella and hepatitis A vaccination.

The randomization list was generated at GSK (Rixensart, Belgium) using MATerial EXcellence (MATEX), a program developed for use with Statistical Analysis Systems (SAS) software. Treatment allocation was performed at the study centers using a central internet-based randomization system with treatment numbers allocated by MMR dose and using a minimization procedure accounting for the center, country, and whole study. Data were collected in an observer-blind manner: enrolled children and their parents or LARs, those responsible for laboratory testing and for the evaluation of study endpoints were all unaware of which vaccine was given. Staff handling the vaccines did not participate in any of the analyses.

Children in stable health, for whom the parents or LARs were expected to comply with protocol requirements and provided written informed consent, who had not been vaccinated against and had no history of measles, mumps, rubella, varicella/zoster, or hepatitis A disease and had not been exposed to measles, mumps, rubella, or varicella/zoster within 30 days before enrollment, were eligible to participate. Children in the US had to have received all routine vaccinations as recommended by the Advisory Committee on Immunization Practices prior to enrollment, including a three-dose infant PCV13 series completed at least 60 days before enrollment. Children in the US who had received a fourth dose of PCV13 were excluded. A complete list of exclusion criteria is provided in the Supplemental methods.

### Study objectives

The study aimed to demonstrate the safety profile of an upper-range release titer MMR-RIT lot compared to commercial MMR II lots when co-administered with varicella and hepatitis A vaccines (in all children) and PCV13 (in children in the US), in terms of fever > 39.0°C (co-primary objective 1) and fever ≥ 38.0°C (co-primary objective 2) within 5–12 days after vaccination. Success criteria for these objectives are detailed in the statistical analysis section. Secondary objectives included the evaluation of safety and reactogenicity of MMR-RIT and MMR II, including the occurrence of measles-like illnesses within 5–12 days after vaccination, and assessment of the immunogenicity of MMR-RIT and MMR II in terms of seroresponse and GMCs for anti-measles, anti-mumps, and anti-rubella virus antibodies at Day 42.

### Reactogenicity and safety assessments

The study staff closely observed the children for at least 30 minutes after vaccination. Reactogenicity and safety were assessed at each visit and through diary cards completed by the parents or LARs. The parents/LARs were given diary cards at the first visit (Day 0) to record solicited symptoms, unsolicited AEs, and concomitant medication/vaccination/treatment up to the second visit (Day 42). Diary cards given at the second visit were used to record any medication/vaccination and any AE occurring between Day 42 and study end. Completed diary cards were returned at the next visit and were verified during discussion with the parents/LARs. Solicited local symptoms (pain, redness, and swelling) at the MMR injection site were recorded for 4 days (Days 0–3) after vaccination. The solicited general symptoms drowsiness, irritability, and loss of appetite were recorded for 15 days (Days 0–14), while fever (defined as a temperature ≥ 38.0°C/100.4°F, preferably measured axillary), and MMR-specific symptoms including rash, parotid/salivary gland swelling, and febrile convulsions were recorded for 43 days (Days 0–42) after vaccination (Figure 4).

Measles-like illnesses were recorded from Day 5 through Day 12 after vaccination and were defined as the occurrence of the following signs or symptoms in the absence of another confirmed diagnosis: temperature ≥ 38.0°C/100.4°F and maculopapular rash (including measles/rubella-like rash) during Days 5–12 post-vaccination, and at least one of the following signs or symptoms: cough, runny nose, conjunctivitis, or diarrhea. Additional information on the recording of rashes, measles-like illnesses, and febrile convulsions is provided in the Supplemental methods.

Unsolicited AEs were recorded for 43 days after vaccination. SAEs, NOCDs (e.g., autoimmune disorders, asthma, type I diabetes, vasculitis, celiac disease, conditions associated with sub-acute or chronic thrombocytopenia, and allergies), and AEs prompting ER visits were recorded throughout the study. The definition of an SAE is provided in the Supplemental methods.

The investigators assessed the intensity of all AEs and their causal relation to vaccination. Definitions of grade 3 intensity for different AEs are included in the Supplemental methods.

### Immunogenicity assessments

Blood samples (approximately 4 mL per child) for immunogenicity assessment were collected on Day 0 (prior to vaccination) and Day 42 (Figure 4). Sera were stored at −20°C until assayed at a designated laboratory. The concentrations of immunoglobulin G (IgG) antibodies against measles and rubella viruses were measured using a commercial enzyme-linked immunosorbent assay (ELISA) kit (*Enzygnost*, Dade Behring) at NÉOMED-LABS Inc. (Laval, Quebec, Canada). Concentrations of IgG antibodies against mumps virus were measured by ELISA at Pharmaceutical Product Development Inc. (Wayne, PA, US). Children were considered seronegative before vaccination if their antibody concentrations were below the following cut-offs: 150 mIU/mL for measles, 5 EU/mL for mumps, and 4 IU/mL for rubella. A seroresponse for the different antigens was defined as a post-vaccination antibody concentration ≥ 200 mIU/mL for measles, ≥ 10 EU/mL for mumps, and ≥ 10 IU/mL for rubella among children who were seronegative before vaccination. These thresholds are accepted by the US Food and Drug Administration as concentrations offering clinical benefit.

### Statistical analyses

A total of 1647 evaluable children (approximately 1098 in the MMR-RIT and 549 in the pooled MMR II group) would give an overall power of 90.37% to meet the two co-primary objectives. Assuming that diary cards of solicited symptoms would not be returned for up to 5% of children, we therefore aimed to enroll 1734 children (approximately 1156 in the MMR-RIT group and 289 each in the two MMR II groups). To keep the type I error below 2.5%, a hierarchical procedure was used for the co-primary objectives; the objective on fever ≥ 38.0°C could only be reached if the objective on fever > 39.0°C was met. The observed rates of fever > 39.0°C or ≥ 38.0°C were considered comparable between the MMR-RIT and MMR II groups (i.e., co-primary objectives were met) if the upper limit of the 2-sided standardized asymptotic 95% CI for the group difference (MMR-RIT minus MMR II) in incidence of fever within 5–12 days after vaccination was ≤ 5% (for fever > 39.0°C) or ≤ 10% (for fever ≥ 38.0°C). These limits were chosen based on the assumption that fever > 39.0°C has a higher clinical impact than fever > 38.0°C and a smaller difference between vaccine groups would therefore be desirable for fever > 39°C than > 38.0°C.

The primary analyses of safety were based on the total vaccinated cohort, which included all children with documented administration of one of the MMR vaccines. Percentages and numbers of children reporting each of the safety outcomes were calculated with exact 95% CIs.

The primary analyses of immunogenicity were based on the ATP cohort for immunogenicity, which included all eligible children who received the study vaccines as per protocol, complied with study procedures, had pre- and post-vaccination serology results available for at least one MMR vaccine antigen and who were below the assay cut-off for at least one MMR vaccine antigen before vaccination. Percentages and numbers of children reaching the predefined immunological thresholds (with exact 95% CIs) and antibody GMCs (with 95% CIs) for each MMR vaccine antigen were calculated. All analyses except for the co-primary objectives were descriptive.

## Abbreviations

AE: adverse event
ATP: according-to-protocol
CCID₅₀: cell culture infectious dose 50
CI: confidence interval
ELISA: enzyme-linked immunosorbent assay
ER: emergency room
EU: ELISA unit
GMC: geometric mean concentration
IgG: immunoglobulin G
IU: international unit
LAR: legally acceptable representative
MATEX: MATerial Excellence
MMR: measles, mumps, rubella
MMR-RIT: GSK’s measles, mumps, rubella vaccine
MMR II: Merck’s measles, mumps, rubella vaccine
NOCD: new onset chronic disease
PCV13: 13-valent pneumococcal conjugate vaccine
SAE: serious adverse event
SAS: Statistical Analysis Systems
TCID_50_: tissue culture infectious dose 50
US: United States

## Trademark statement

*Priorix* and *Havrix* are trademarks of the GSK group of companies. *M-M-R II* and *Varivax* are trademarks of Merck & Co., Inc. *Prevnar 13/Prevenar 13* is a trademark of Wyeth LLC, marketed by Pfizer Inc.
