# Supplementary material for: Safety and immunogenicity of an upper-range release titer measles-mumps-rubella vaccine in children vaccinated at 12 to 15 months of age: a phase III, randomized study
Source: Hum Vaccin Immunother. 2018 Aug 29;14(12):2921–31. doi: 10.1080/21645515.2018.1502527 (PMC6343620; doi:10.1080/21645515.2018.1502527)
Supplement: Supplemental Material [file khvi-14-12-1502527-s001.docx]

**Supplemental online material**

**Supplemental methods**

Exclusion criteria

- Child in care, i.e., a child placed under the control or protection of an agency, organization, institution or entity by the courts, the government, or a government body, acting in accordance with powers conferred on them by law or regulation. This includes children cared for by foster parents or living in a care home, but does not include children who are adopted or have been appointed a legally acceptable representative (LAR).
- Use of any investigational or non-registered product (drug or vaccine) other than the study vaccine(s) during the period starting 30 days before the day of study vaccination (Day 0) or planned use during the entire study period.
- Concurrently participating in another clinical study, in which the child had been or would be exposed to an investigational or a non-investigational product (pharmaceutical product or device).
- Chronic administration (defined as 14 or more consecutive days) of immunosuppressants, or other immune-modifying drugs during the period starting 180 days prior to the study vaccination on Day 0 or any planned administration of immunosuppressive and immune-modifying drugs during the entire study.
  - For corticosteroids, this meant prednisone ≥0.5 mg/kg/day or equivalent.
  - Inhaled and topical steroids are allowed.
- (Planned) administration of a vaccine not foreseen by the study protocol during the period starting 30 days prior to the study vaccination on Day 0 and ending at the second visit (Day 42).
  - Inactivated influenza vaccine and monovalent *Haemophilus influenzae* type b conjugate vaccine could be given at any time, including the day of study vaccination. These vaccines had to be administered at a different location than the study vaccine(s).
  - Any other age-appropriate vaccine could be given starting at the second visit (Day 42) and anytime thereafter.
- Administration of immunoglobulins and/or any blood products during the period starting 180 days before the study vaccination on Day 0 or planned administration from the date of vaccination through the immunogenicity evaluation at the second visit (Day 42).
- History of measles, mumps, rubella, varicella/zoster, and/or hepatitis A disease.
- Known exposure to measles, mumps, rubella, and/or varicella/zoster during the period starting within 30 days prior to the first study vaccination.
- Previous vaccination against measles, mumps, rubella, hepatitis A, and/or varicella virus.
- Any confirmed or suspected immunosuppressive or immunodeficient condition, based on medical history and physical examination (no laboratory testing required).
- Blood dyscrasias, leukemia, lymphomas of any type, or other malignant neoplasms affecting the bone marrow or lymphatic systems.
- A family history of congenital or hereditary immunodeficiency.
- History of allergic disease or reactions likely to be exacerbated by any component of the vaccines, including hypersensitivity to neomycin, latex, or gelatin.
- Acute disease at the time of enrollment. Acute disease was defined as the presence of a moderate or severe illness with or without fever. Fever was defined as a temperature ≥38.0°C/100.4°F by any age-appropriate route. All vaccines could be administered to persons with a minor illness such as diarrhea or mild upper respiratory infection without fever.
- Active untreated tuberculosis based on medical history.
- Any other condition which, in the opinion of the investigator, prevented the child from participating in the study.
- For children enrolled in the US only: a child that previously received a fourth dose of 13-valent pneumococcal conjugate vaccine.

Recording of rashes and measles-like illness

If any fever ≥38.0°C/100.4°F occurred during Days 0–42, the parents/LARs had to contact the study site. If fever occurred during Days 5–12 after vaccination and a rash was also present that the investigator suspected could be measles-like, the investigator inquired for additional signs and symptoms (cough, runny nose, conjunctivitis, or diarrhea) to assess if a potential measles-like illness had occurred as a result of vaccination. A visit was arranged ideally within 48 hours after rash onset and follow-up calls were made to assess the evolution of signs and symptoms.

Parent(s)/LAR(s) were provided with a description of rashes and were asked to contact the study site immediately if their child developed a potential measles/rubella or varicella-like rash during Days 0–42. If the investigator also suspected a measles/rubella or varicella-like rash, a visit was arranged ideally within 48 hours after rash onset. If fever was reported with the rash during Days 5–12 and the investigator suspected a potential measles-like rash, the investigator inquired for additional signs or symptoms to assess a potential measles-like illness.

Rashes were classified as:

(i) Measles/rubella-like rash (macular or maculopapular rashes): presence of macules, discolored small patches or spots of the skin, neither elevated nor depressed below the skin's surface and/or papules, raised bumps on the skin usually <1 cm in diameter.

(ii) Varicella-like rash (papulovesicular): simultaneous presence of papules and vesicles raised above the skin's surface.

(iii) Other type of rash (e.g., heat rash).

The duration, intensity, location, causality, and outcome of the rash were assessed.

Recording of febrile convulsion

If a child experienced febrile convulsions (or any other neurological signs or symptoms) during Days 0–42, the parent(s)/LAR(s) had to contact the investigator immediately for follow-up. The investigator classified the level of diagnostic certainty of febrile convulsions and other seizures according to the Brighton Collaboration Seizure Working Group’s case definitions of generalized convulsive seizures.^1^ Febrile convulsions were also classified as simple or complex based on guidelines from the American Academy of Pediatrics.^2^

Grade 3 intensity of adverse events

Grade 3 intensity for the different adverse events (AEs) was defined as crying when the limb was moved or the limb was spontaneously painful (pain), diameter >20 mm (redness, swelling), temperature >39.5°C/103.1°F (fever), >150 lesions (measles/rubella and varicella-like rash), rash preventing normal activity (other rashes), swelling with accompanying general symptoms (parotid/salivary gland swelling), crying inconsolably or preventing normal activity (irritability), not eating at all (loss of appetite), preventing normal activity (all other AEs).

Definition of a serious adverse event (SAE)

An SAE was defined as any untoward medical occurrence that:

(i) resulted in death

(ii) was life-threatening (Note, this referred to an event in which the child was at risk of death at the time of the event and did not refer to an event which hypothetically might have caused death, had it been more severe.)

(iii) required hospitalization or prolongation of existing hospitalization (Note, this meant that the child was admitted at the hospital or emergency ward for observation and/or treatment that would not have been appropriate in the physician’s office or in an out-patient setting. Complications that occurred during hospitalization were also considered AEs. If a complication prolonged hospitalization or fulfilled any other serious criteria, the event was also considered serious. In case of doubt as to whether ‘hospitalization’ occurred or was necessary, the AE was considered serious. Hospitalization for elective treatment of a pre-existing condition—known or diagnosed prior to informed consent signature—that did not worsen from baseline was not considered an AE.)

d. resulted in disability/incapacity (Note, the term disability meant a substantial disruption of a person’s ability to conduct normal life functions. This definition was not intended to include experiences of relatively minor medical significance such as uncomplicated headache, nausea,

vomiting, diarrhea, influenza like illness, and accidental trauma—such as a sprained ankle—which may have interfered with or prevented everyday life functions but did not constitute a substantial disruption.)

**Supplemental table S1. Incidence of solicited injection site and general symptoms (total vaccinated cohort)**

| **Symptom** |  | **MMR-RIT**  **N=1126*** | |  | **MMR II**  **N=555*** | |  |  |
| --- | --- | --- | --- | --- | --- | --- | --- | --- |
|  |  | **n** | **% (95% CI)** |  | **n** | **% (95% CI)** | |  |
| **Injection site symptoms (Days 0–3)** | | | | | | | |  |
| **Pain** | | Any | 312 | 27.8 (25.2, 30.5) |  | 131 | 23.7 (20.2, 27.5) | |
|  | | Grade 3 | 6 | 0.5 (0.2, 1.2) |  | 2 | 0.4 (0.0, 1.3) | |
| **Redness** | | Any | 260 | 23.2 (20.7, 25.7) |  | 137 | 24.8 (21.2, 28.6) | |
|  | | >20 mm | 8 | 0.7 (0.3, 1.4) |  | 7 | 1.3 (0.5, 2.6) | |
| **Swelling** | | Any | 96 | 8.5 (7.0, 10.3) |  | 58 | 10.5 (8.1, 13.3) | |
|  | | >20 mm | 3 | 0.3 (0.1, 0.8) |  | 2 | 0.4 (0.0, 1.3) | |
| **General symptoms (Days 0–14)** | | | | | | | |  |
| **Drowsiness** | | Any | 527 | 46.8 (43.9, 49.8) |  | 238 | 42.9 (38.7, 47.1) | |
|  | | Grade 3 | 31 | 2.8 (1.9, 3.9) |  | 13 | 2.3 (1.3, 4.0) | |
| **Irritability** | | Any | 722 | 64.1 (61.2, 66.9) |  | 345 | 62.2 (58.0, 66.2) | |
|  | | Grade 3 | 42 | 3.7 (2.7, 5.0) |  | 19 | 3.4 (2.1, 5.3) | |
| **Loss of appetite** | | Any | 493 | 43.8 (40.9, 46.7) |  | 232 | 41.8 (37.7, 46.0) | |
|  | | Grade 3 | 20 | 1.8 (1.1, 2.7) |  | 10 | 1.8 (0.9, 3.3) | |
| **General symptoms (Days 0–42)** | | | | | | | | |
| **Fever** | | ≥38.0°C | 350 | 31.1 (28.4, 33.9) |  | 179 | 32.3 (28.4, 36.3) |  |
|  | | >39.5°C | 45 | 4.0 (2.9, 5.3) |  | 15 | 2.7 (1.5, 4.4) | |

N, number of children with documented dose (*for injection site symptoms: N=1123 for MMR-RIT and N=553 for MMR II); n and %, number and percentage of children reporting the specified symptom at least once during the indicated post-vaccination period; CI, confidence interval. Grade 3 intensity was defined as crying when the limb was moved or the limb was spontaneously painful (pain), event preventing normal activity (drowsiness), crying inconsolably or preventing normal activity (irritability), not eating at all (loss of appetite). Injection site symptoms were those recorded at the MMR injection site.

**Supplemental references**

1. Bonhoeffer J, Menkes J, Gold MS, de Souza-Brito G, Fisher MC, Halsey N, Vermeer P, The Brighton Collaboration Seizure Working Group. Generalized convulsive seizure as an adverse event following immunization: case definition and guidelines for data collection, analysis, and presentation. Vaccine. 2004; 22(5-6):557-62.
2. Steering Committee of Quality Improvement and Management, Subcommittee on Febrile Seizures. Febrile seizures: Clinical practice guideline for the long-term management of the child with simple febrile seizures. Pediatrics. 2008;121(6):1281-6. doi: 10.1542/peds.2008-0939
